# Supplementary material for: Chromothripsis during telomere crisis is independent of NHEJ, and consistent with a replicative origin
Source: Genome Res. 2019 May;29(5):737–49. doi: 10.1101/gr.240705.118 (PMC6499312; doi:10.1101/gr.240705.118)
Supplement: Supplemental Material [file supp_gr.240705.118_Supplemental_file_1.zip › contigs/annotated_contigs/DB71/contig.2.DB71_length_468_mean_cov_13.297008547.docx]

**DB71_length_468_mean_cov_13.297008547**

```
ATGTTCTATTAGTATTTGCAAAGTAAGAAAGTCAAGTCATTATGAAATGATATCCTATTAACTTACATTAATGAAAGTGACACAGCATA
 >chr8:20901471-20901900 - E=1e-224 p=0e+00
AAGTCATTCCTCTTGGTCCAGACATTGCCCCAGCCTGCCTGGTGGTGCTACCTGGTACCCAAGCACATCTCCAGCTCCCACTCTGTGCT

TGGAGGGGTGCAGTGAAGACATGGCACAGGTTCCAGGTACCATCAGCTGAAGATTCCAACCAGGTGGGAGGTAGGAGGTGGGATAGTGC

AAGGTGGGCTTGGAGGATGGTGGGGAGGAGGTGGGCTTGGTGGATGATGGGAAGGAGATGGGGTGGTGGAAGGTGGGGAGGAGGTGGGG

TGGTGGAAGGTGGGGGGGAGGTGGGGGGGTGGAAGGTGGGGGGGAGGTGGGGGGGTGGAAGGTGGGGGGG|G|GGGGGGGGTGGGGGGA
                                                                        >chr15:78285182
GGTGGGGGGGGAGTGGGGGGG|TGGA-78285219 - E=1e-01
```
